# Exploring abnormal Cambrian-aged trilobites in the Smithsonian collection

**DOI:** 10.7717/peerj.8453

**Published:** 2020-02-03

**Authors:** Russell D.C. Bicknell, Stephen Pates

**Affiliations:** 1 Palaeoscience Research Centre, School of Environmental and Rural Science, University of New England, Armidale, NSW, Australia; 2 Museum of Comparative Zoology and Department of Organismic and Evolutionary Biology, Harvard University, Boston, United States of America

**Keywords:** Trilobites, Abnormalities, Cambrian Explosion, Durophagy, Escalation, Healed injuries

## Abstract

Biomineralised trilobite exoskeletons provide a 250 million year record of abnormalities in one of the most diverse arthropod groups in history. One type of abnormality—repaired injuries—have allowed palaeobiologists to document records of Paleozoic predation, accidental damage, and complications in moulting experienced by the group. Although Cambrian trilobite injuries are fairly well documented, the illustration of new injured specimens will produce a more complete understanding of Cambrian prey items. To align with this perspective, nine new abnormal specimens displaying healed injuries from the Smithsonian National Museum of Natural History collection are documented. The injury pattern conforms to the suggestion of lateralised prey defence or predator preference, but it is highlighted that the root cause for such patterns is obscured by the lumping of data across different palaeoecological and environmental conditions. Further studies of Cambrian trilobites with injuries represent a key direction for uncovering evidence for the Cambrian escalation event.

## Introduction

The Cambrian Explosion—a rapid and stunning increase in animal diversity and disparity during the earliest Paleozoic, over 500 million years ago—likely resulted from a combination of biological, ecological, and environmental factors (Smith & Harper, 2013; Zhang et al., 2014; Bicknell & Paterson, 2018). Among the ecological factors, the rise of predation has been considered a key evolutionary innovation that helped drive and shape morphological and diversity trajectories of different Cambrian groups (Vermeij, 1989; Conway Morris, 1998; Bengtson, 2002; Babcock, 2003; Wood & Zhuravlev, 2012; Bicknell & Paterson, 2018; Pates & Bicknell, 2019), including the rise of biomineralisation in the exoskeletons and shells of prey animals (Vermeij, 1989; Vermeij, 2013; Conway Morris & Jenkins, 1985; Babcock, 1993; Babcock, 2003; Conway Morris & Bengtson, 1994; Bicknell & Paterson, 2018). This biomineralisation also permitted the documentation of failed predation events, complications during moulting, as well as genetic, developmental and/or behavioural malfunctions in trilobites and other animals (Owen, 1985; Babcock, 1993; Babcock, 2007; Bicknell & Paterson, 2018).

Trilobites were an abundant, diverse, and disparate group of Paleozoic animals with biomineralised exoskeletons (Webster, 2007) that commonly record abnormal features. A range of Cambrian-aged trilobite abnormalities are known and have been used as a model system for exploring Cambrian predator–prey interactions (Owen, 1985; Conway Morris & Jenkins, 1985; Babcock & Robison, 1989; Babcock, 1993; Babcock, 2003; Lee, Choi & Pratt, 2001; Pates et al., 2017; Bicknell & Paterson, 2018; Vinn, 2018; Pates & Bicknell, 2019). Here, nine new examples of abnormal specimens from the Paleontological collection of the Smithsonian National Museum of Natural History (USNM PAL) are presented; a collection from which abnormal trilobites have previously been reported (Babcock, 1993; Bicknell, Pates & Botton, 2018d). These specimens aid the recent pulse in documentation of Cambrian predation traces and other abnormalities (Fatka, Budil & Grigar, 2015; Pates et al., 2017; Bicknell, Pates & Botton, 2018d; Bicknell & Pates, 2019; Klompmaker et al., 2019; Pates & Bicknell, 2019).

## Methods

Cambrian-aged trilobite specimens in the USNM PAL were reviewed for evidence for abnormalities, as originally defined by Owen (1985). This definition has subsequently been used and further refined in studies of other abnormal arthropods (Babcock, 1993; Bicknell & Paterson, 2018; Bicknell & Pates, 2019; see section ‘Terminology’). Specimens were photographed using a Canon EOS REBEL T2i under LED (light-emitting diode) lighting. Measurements of abnormalities were made from photographs using ImageJ (version 1.52a; Schneider, Rasband & Eliceiri, 2012). Specimen ages were determined from the age of the host rock and comparisons to the literature, using the most recent geological timescale (Peng, Babcock & Cooper, 2012).

### Terminology

#### Cicatrisation

Thickening of the exoskeleton over an injury which occurred in same inter-moult period that the injury was sustained (Ludvigsen, 1977; Owen, 1985).

#### Injuries

Abnormal features that record unsuccessful predation, complications during burrowing, mating, or moulting (Bicknell, Pates & Botton, 2018d). They are indicated by exoskeletal repair, substantial exoskeletal deformity. They can have ‘U’-, ‘V’-, ‘W’-, or ‘L’-shapes impacting multiple sections of the exoskeleton, or be expressed as single segment injuries (SSIs): the shortening and possible rounding of the distal margin of singular thoracic segments (formally referred to as single spine injuries, Pates & Bicknell, 2019).

#### Regeneration

Regrowth of an injured area, over a series of moults (Owen, 1985; Pates et al., 2017).

## Results

**Figure 1:**
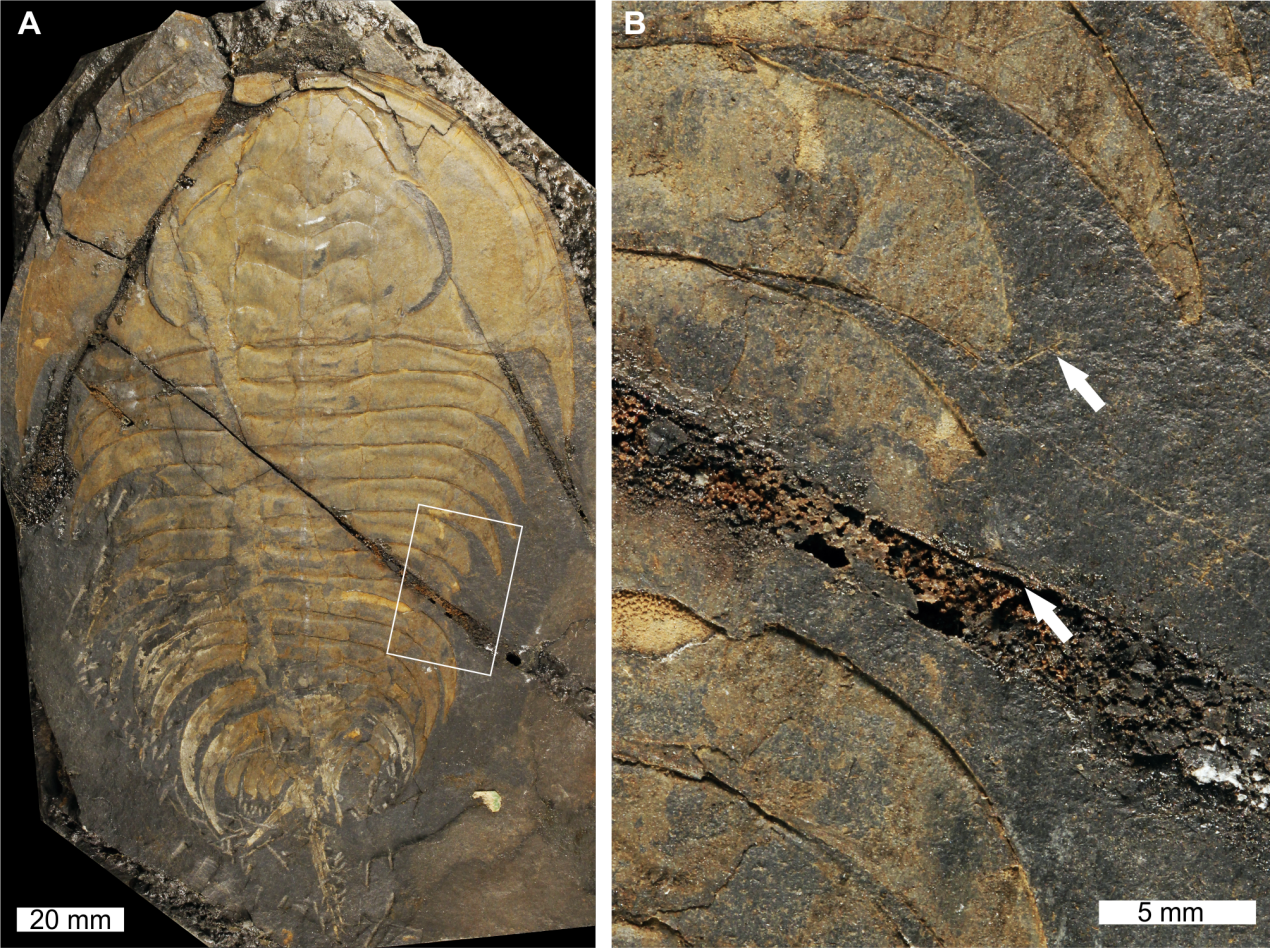
*Elliptocephala asaphoides*Emmons, 1844, USNM PAL 18350a, Browns Pond Formation (Cambrian Stage 2, Series 4). (A) Complete specimen. (B) Close up of abnormality in box in (A) illustrating pleural truncation and slight ‘U’-shaped injury (white arrows).

*Elliptocephala asaphoides* Emmons, 1844, USNM PAL 18350a, Browns Pond Formation (=Schodack Formation) (Cambrian Stage 2, Series 4, age taken from Skovsted & Peel, 2007), eastern New York, USA. Figures 1A, 1B.

USNM PAL 18350a is preserved as an external mould with a ‘U’-shaped abnormality that truncates pleurae by 8 mm. Abnormality begins at thoracic segment 6, extends into thoracic segment 8, and is 15 mm long. The margin of the abnormality is cicatrised along thoracic segment 6, while thoracic segments 7 and 8 show no evidence for cicatrisation.

**Figure 2:**
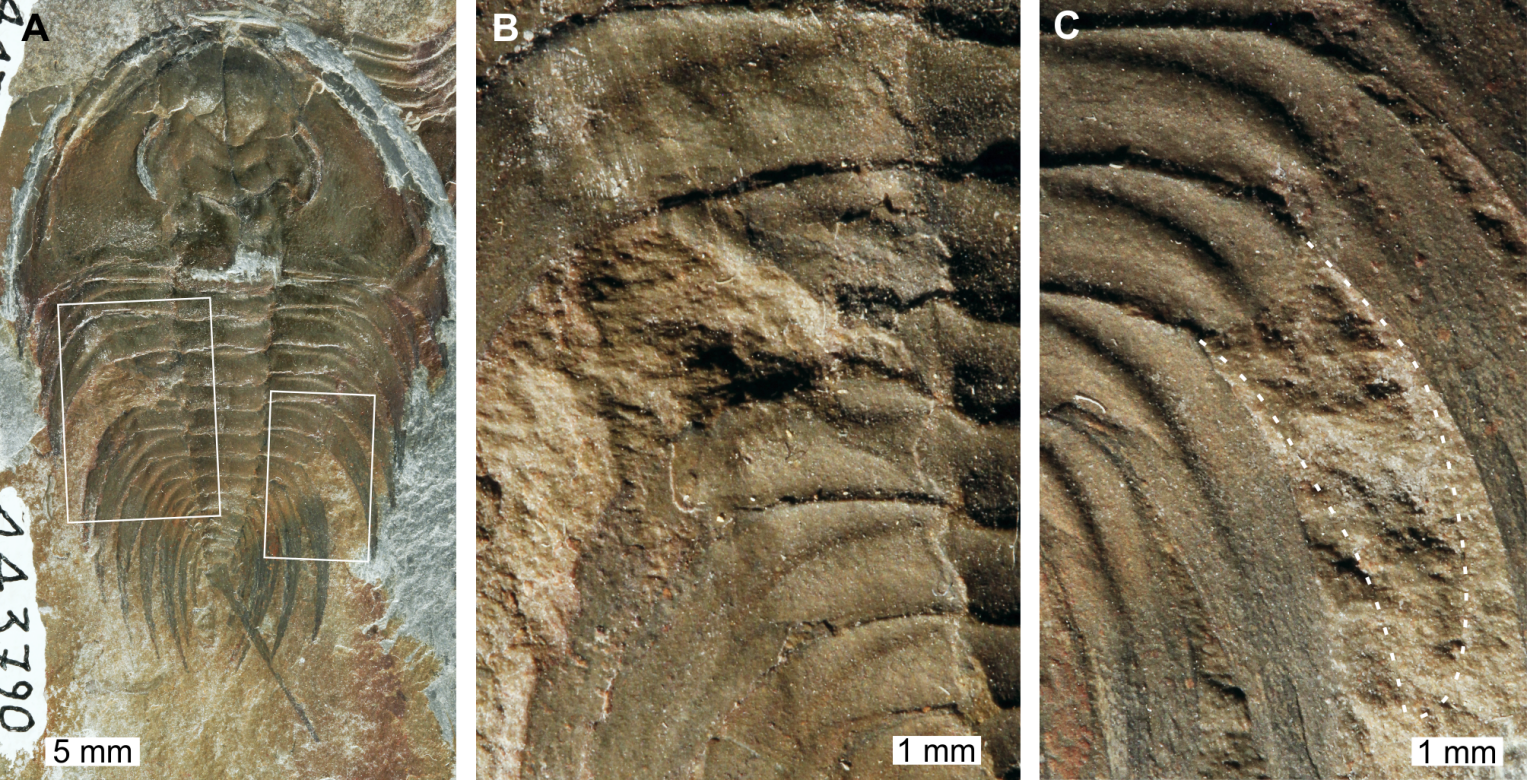
*Mummaspis oblisooculatus*Fritz, 1992*,* USNM PAL 443790, Mural Formation (Cambrian Series 2, Stage 4)*.* Specimen was figured in Fritz (1992, pl. 17, fig. 4). (A) Complete specimen showing an injury and evidence for taphonomic alteration. (B) Close up of abnormality on the left side of the thorax illustrating pleural truncation and ‘V’-shape. (C) Close up broken thoracic segment on the on the right thoracic lobe. Dotted white line shows where part of a spine is preserved.

*Mummaspis oblisooculatus* Fritz, 1992, USNM PAL 443790, Mural Formation (Cambrian Series 2, Stage 4, age taken from Ortega Hernández, Esteve & Butterfield, 2013), Alberta, Canada. Figures 2A–2C.

USNM PAL 443790 is preserved as an external mould and displays a possible bilateral thoracic abnormality. Thoracic segments 3 and 4 on the left side have been truncated into an asymmetric ‘V’-shaped abnormality that is 3 mm long and slightly cicatrised (Fig. 2B). The thoracic segments are truncated pleurae by 9 mm. On the right side of the thorax, there is a potential SSI on thoracic segment 6 (Fig. 2C). However, closer examination of the specimen highlights that there are likely traces of more parts of the pleural spine. In this case, this feature reflects taphonomic alteration to the specimen, or poor breakage of the rock.

**Figure 3:**
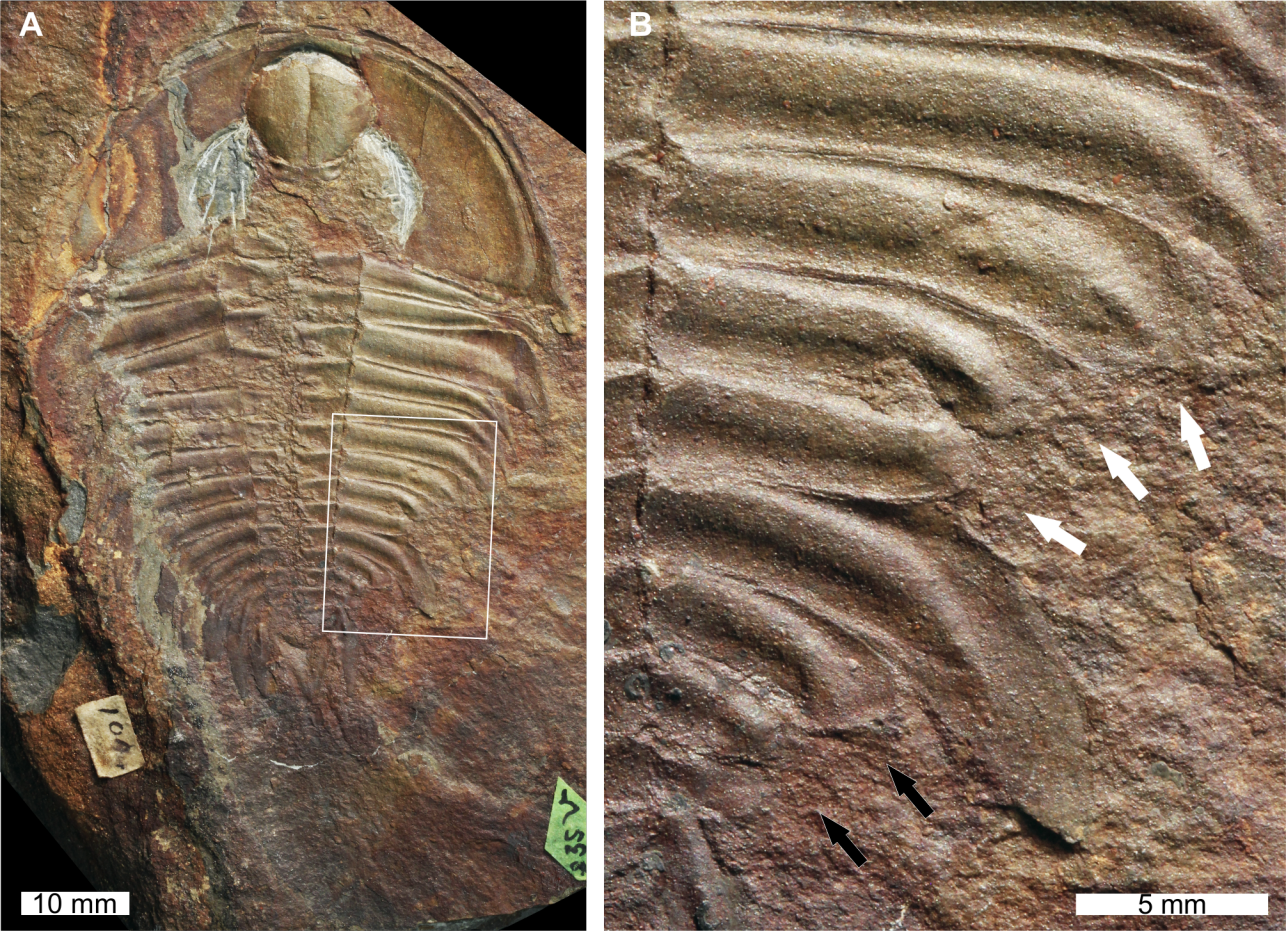
*Olenellus thompsoni* (Hall, 1859) *sensu*Lieberman (1999).** UNSM PAL 729428, Parker Formation (Cambrian Stage 2, Series 4). (A) Complete specimen. (B) Close up of abnormalities in box in (A). The anterior ‘U’-shaped abnormality (white arrows) and the posterior ‘W’-shaped abnormality (black arrows).

*Olenellus thompsoni* (Hall, 1859), USNM PAL 729428, Parker Formation (Cambrian Stage 2, Series 4, age taken from Webster & Landing, 2016), Vermont, USA. Figures 3A, 3B.

USNM PAL 729428 is preserved as an external mould with little relief and shows two abnormalities on the right posterior thorax. The more anterior abnormality has a ‘U’-shape, is observed on thoracic segments 7–9, and is slightly cicatrised. Thoracic pleurae 7 and 8 are fused together at the abnormality margin. The second abnormality is ‘W’-shaped, spans thoracic segments 11–13, and has a cicatrised margin. Both abnormalities truncate the thoracic pleurae by 6 mm.

**Figure 4:**
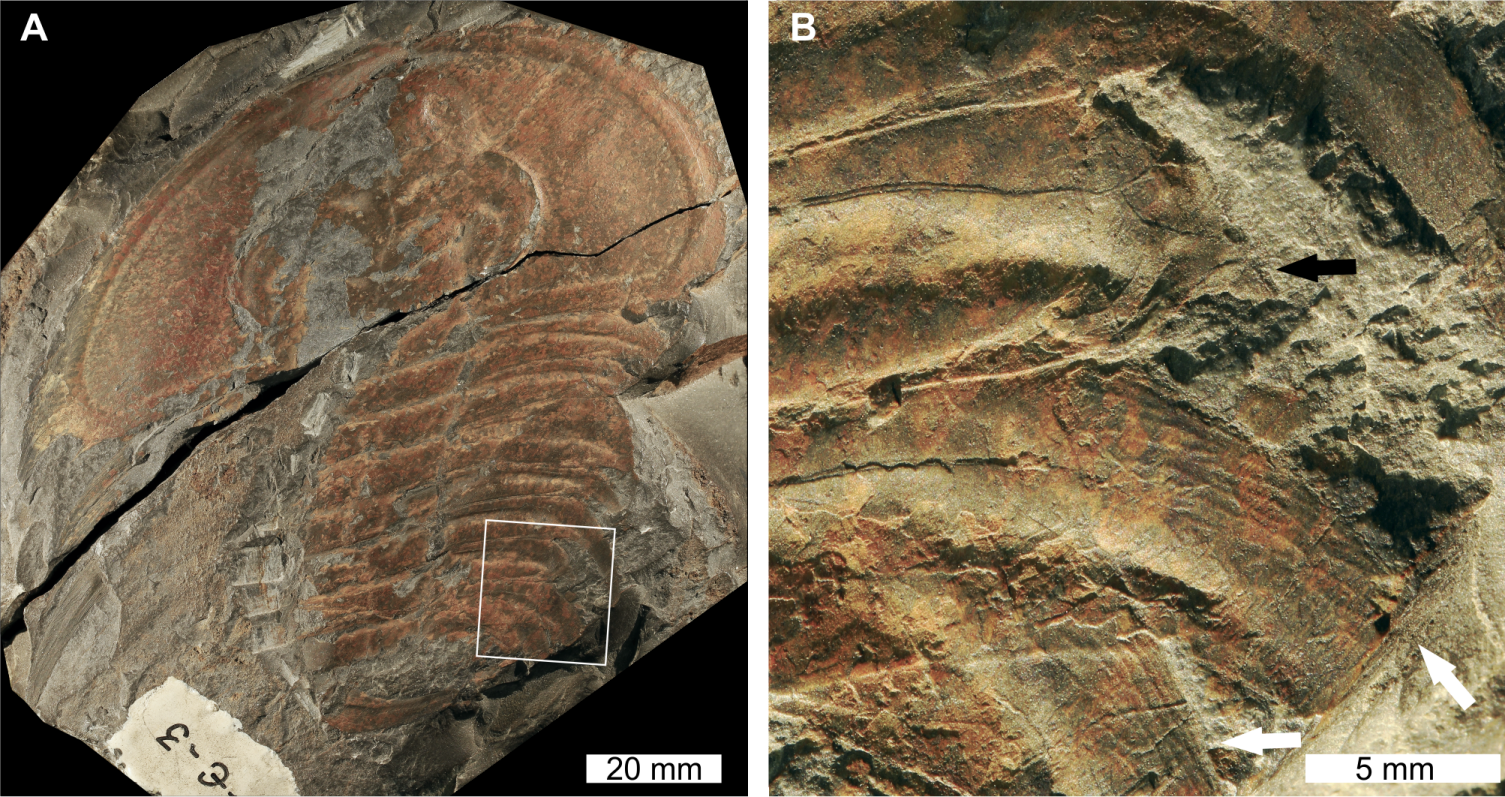
*Olenellus getzi*Dunbar, 1925, UNSM PAL 729422, Kinzers Formation (Cambrian Series 2, Stage 4). (A) Complete specimen. (B) Close up of abnormalities in box in (A). The SSI on pleura 6 (black arrow) and the divergent spine on pleura 7 (white arrows).

*Olenellus getzi* Dunbar, 1925, USNM PAL 729422**,** Kinzers Formation (Cambrian Series 2, Stage 4), Pennsylvania, USA. Figures 4A, 4B.

USNM PAL 729422 is a partial specimen preserved as an external mould and displays two abnormalities on the right side of the thorax. The more anterior abnormality is an SSI on the 6th thoracic segment that shows no evidence of cicatrisation and truncates the pleura by 6 mm. Thoracic segment 7 shows possible evidence of two thoracic pleurae developing from the one thoracic segment. The split between these spines occurs ∼24 mm from the midline of the specimen. As the upper of the two pleurae shows terraced lines indicative of the ventral surface (Lieberman, 1999), it is possible that this specimen instead represents a fragment retained during moulting, or even the chance superimposition of a fragment on a complete specimen. Fragments on the specimen indicate that either of these two scenarios is possible; however, the alignment of the pleura with the segment, and its relative size, support a biological interpretation.

**Figure 5:**
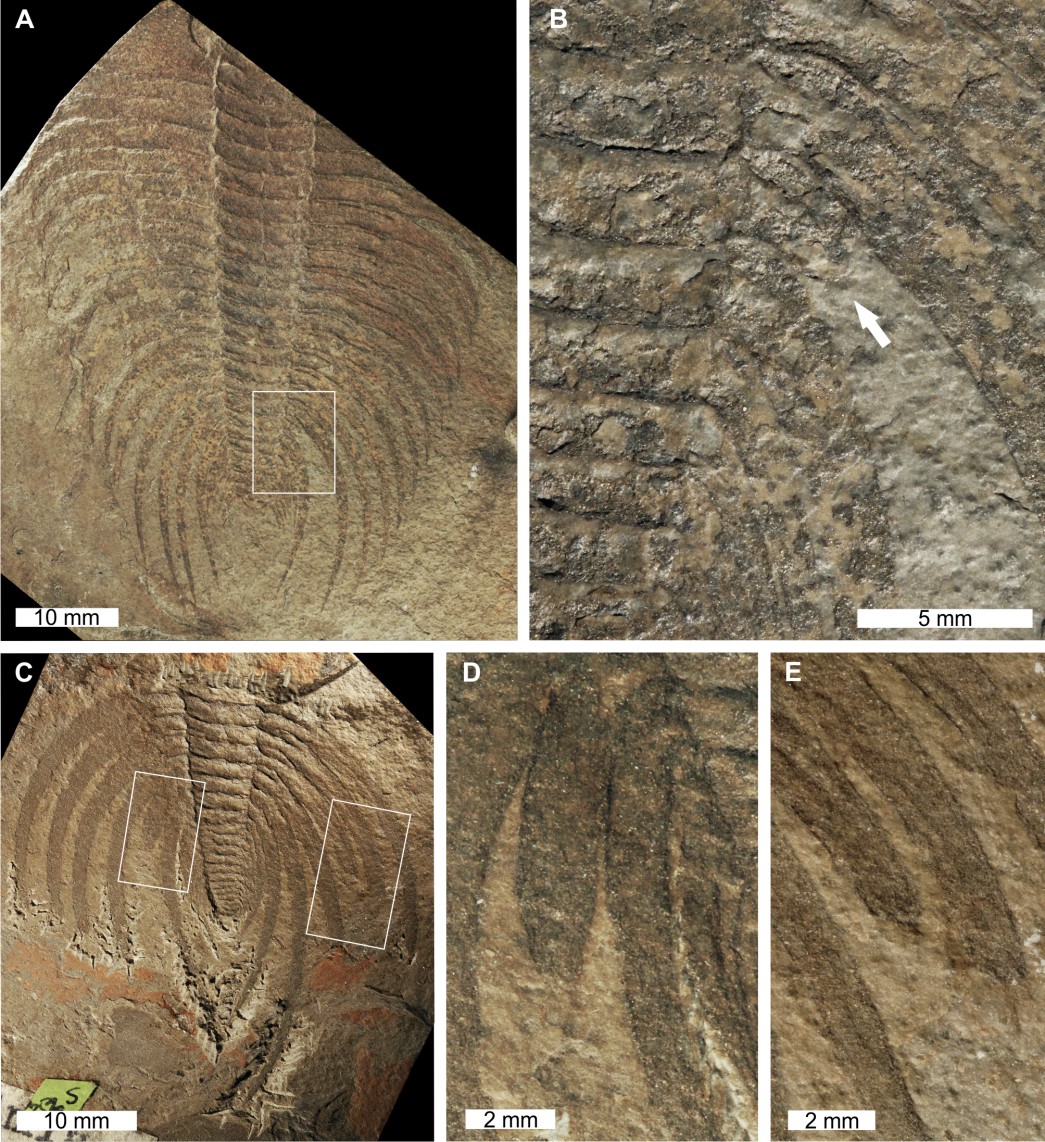
*Nevadia weeksi*Walcott, 1910, USNM PAL 56792a; USNM PAL 56792d, Pioche Formation (Miaolingian Series, Wuliuan). Specimens were originally figured in Walcott (1910, pl. 23, figs 1, 4) and Whittington (1989, figs. 47, 49). (A) Complete specimen of USNM PAL 56792a. (B) Close up of abnormality in box in (A) illustrating SSI on 16th thoracic segment (white arrow). (C) Complete specimen of USNM PAL 56792d (D) Close up of left box in (C) showing truncated 14th thoracic segment. (E) Close up of right box in (C) showing thoracic segments 11th and 12th with SSIs.

*Nevadia weeksi* Walcott, 1910, USNM PAL 56792a; USNM PAL 56792d, Pioche Formation (Miaolingian Series, Wuliuan, age taken from Kimmig, Meyer & Lieberman, 2018), Utah, USA Figs. 5A–5E.

USNM PAL 56792a is a partial specimen preserved as an external mould with an abnormality on the posterior right thorax (Figs. 5A, 5B). The abnormality is an SSI on the 16th thoracic segment. The pleura is terminated 2 mm from the thoracic axial lobe, rounded, and shows no evidence of cicatrisation. This abnormality truncates the pleura by 28 mm.

USNM PAL 56792d preserves the posterior section of the exoskeleton and has a bilaterally expressed injury. The plural spine on the 14th thoracic segment on the left side is an SSI that truncates the pleura by 10 mm (Fig. 5D). On the right thoracic side, the 11th and 12th segments show SSIs. The terminus of the 11th thoracic pleura is not rounded and truncated by at least 7 mm (Fig. 5E). The terminus of the 12th thoracic pleura is slightly rounded and truncated by at least 9 mm (Fig. 5E). No abnormalities on this specimen show evidence of cicatrisation.

**Figure 6:**
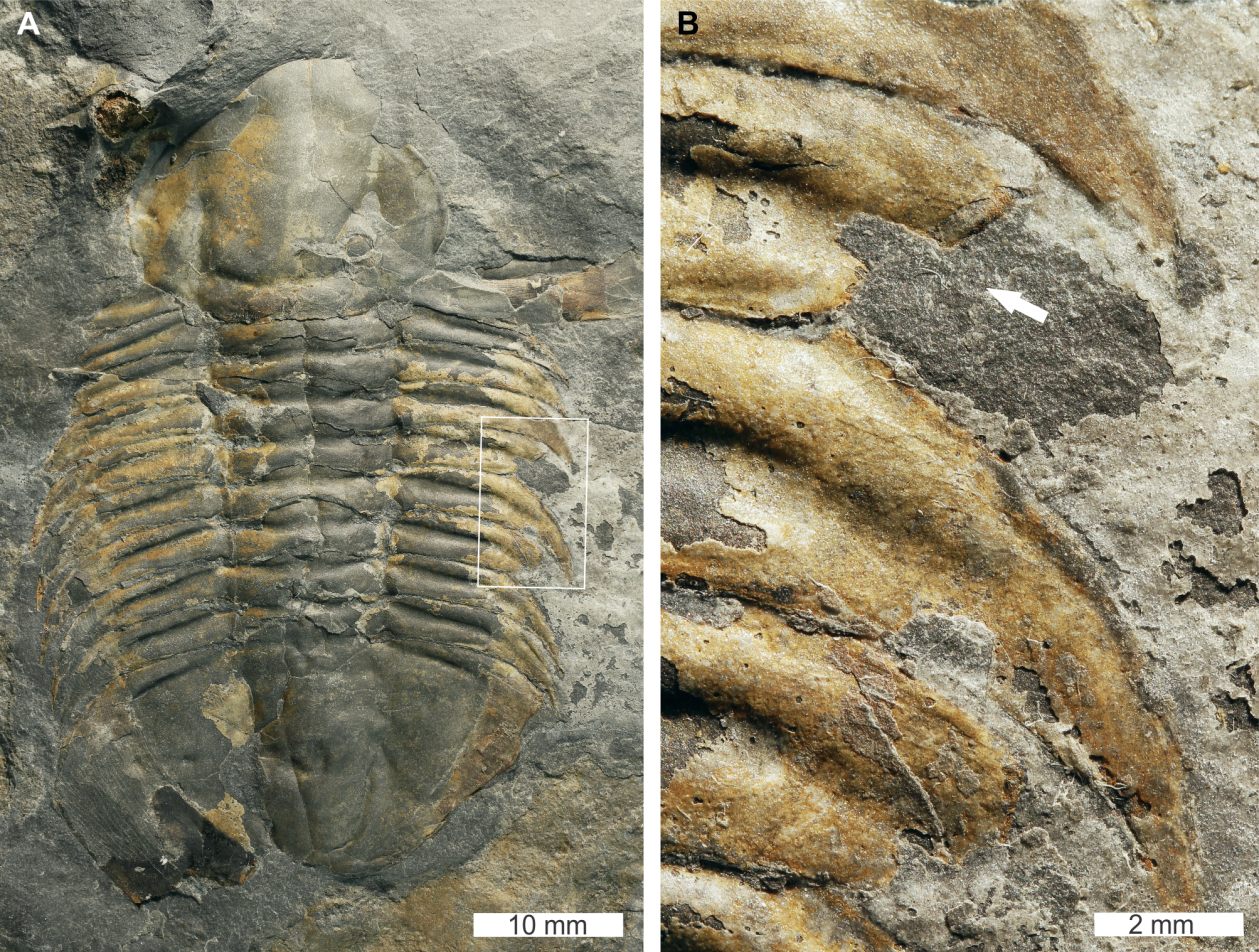
*Glossopleura gigantea*Resser, 1939, UNSM PAL 729419, Spence Shale Member, Langston Formation (Miaolingian Series, Wuliuan). (A) Complete specimen. (B) Close up of abnormality in box in (A) illustrating SSI on 4th thoracic segment (white arrow).

*Glossopleura gigantea* Resser, 1939, USNM PAL 729419, Spence Shale Member, Langston Formation (Miaolingian Series, Wuliuan), Utah, USA Figs. 6A, 6B.

USNM PAL 729419 is preserved as an external mould and displays an abnormal right thorax. Abnormality is an SSI on the 4th thoracic segment that truncates the segment by 8 mm. The margin of the abnormality is rounded and slightly cicatrised.

**Figure 7:**
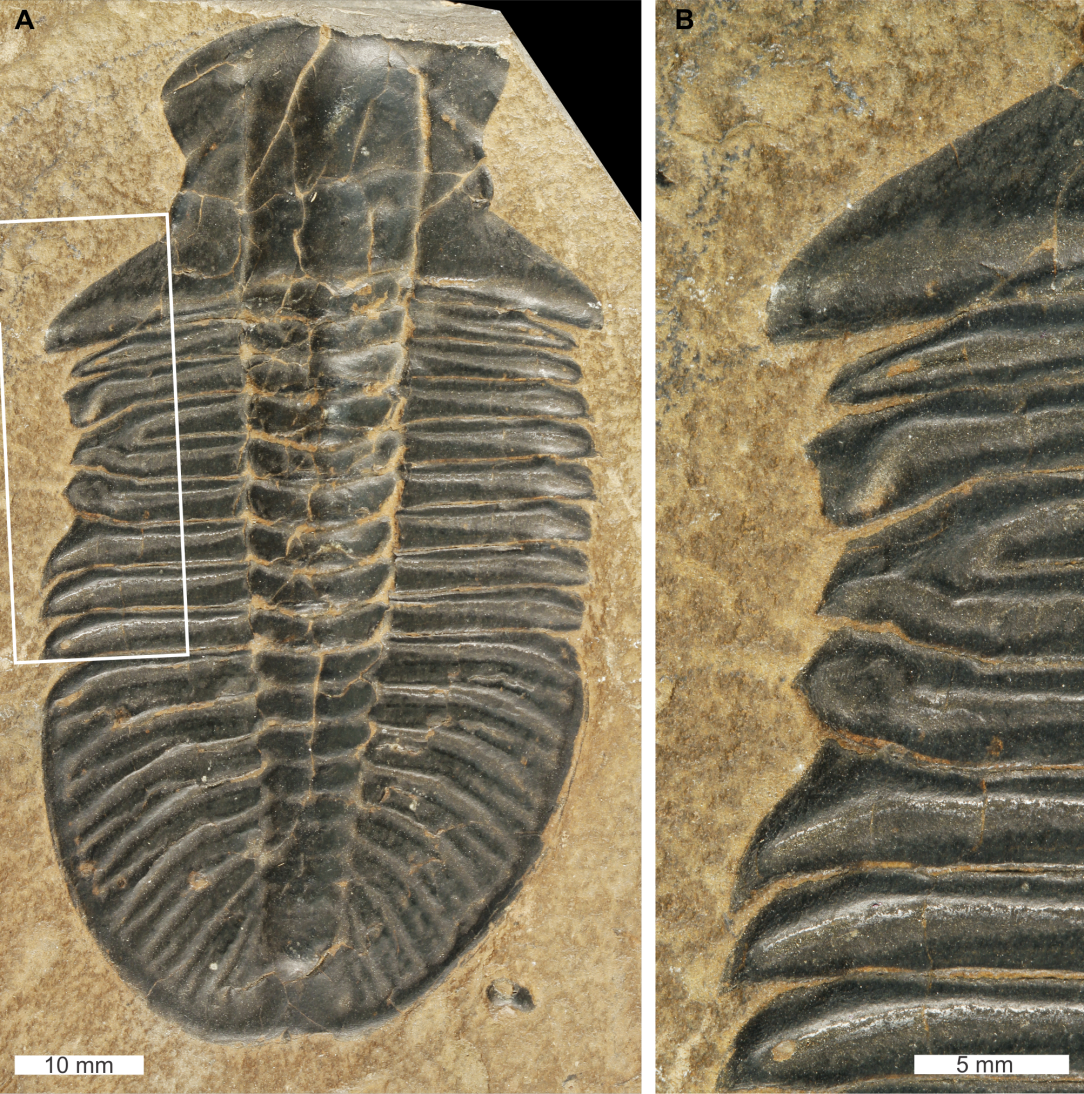
*Ogygopsis klotzi* (Rominger, 1887)*,* UNSM PAL 729421, Stephen Formation (Miaolingian Series, Wuliuan). (A) Complete specimen. (B) Close up of ‘W’-shaped abnormality in box in (A).

*Ogygopsis klotzi* (Rominger, 1887), USNM PAL 729421, Stephen Formation (Miaolingian Series, Wuliuan), British Columbia, Canada. Figures 7A, 7B.

USNM PAL 729421 is preserved as an external mould and displays an abnormality on the left thoracic lobe. The abnormality has a shallow ‘W’-shape that begins at the 2nd thoracic segment, ends at ends at the 5th thoracic segment, is 10.5 mm long, and truncates the pleurae by 2 mm. Abnormality margin shows no evidence of cicatrisation. Thoracic pleurae 3 and 4 are fused together at the abnormality margin, while the margins of pleurae 2 and 5 are distorted about the fused section.

**Figure 8:**
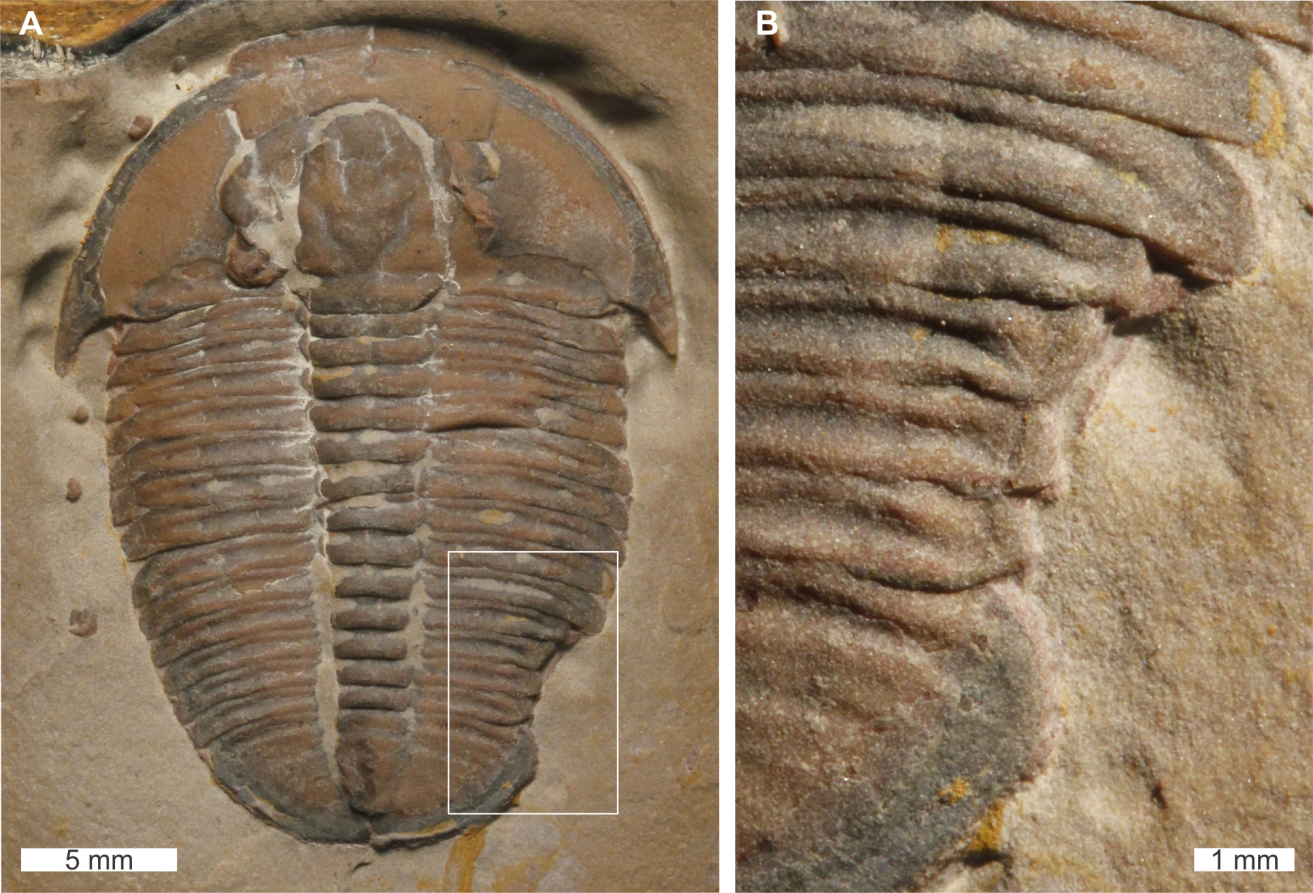
*Elrathia kingii* (Meek, 1870)*,* UNSM PAL 729417, Wheeler Formation (Miaolingian Series, Drumian). (A) Complete specimen. (B) Close up of cicatrised, ‘U’-shaped abnormality in box in (A).

*Elrathia kingii* Meek, 1870, USNM PAL 729417, Wheeler Formation (Miaolingian Series, Drumian), western Utah, USA. Figures 8A, 8B.

USNM PAL 729417 is preserved as an external mould and displays an abnormality on the posterior right thorax that extends into the anterior pygidium. The abnormality has a ‘U’-shape, begins at the 10th thoracic segment, ends within the first 1 mm of the pygidium, is 4.6 mm long, and truncates the affected thoracic segments by 1.5 mm. The abnormality margin is slightly cicatrised and deforms thoracic segments 10–11.

## Discussion

Comparing the nature of the abnormalities documented here with examples in other publications suggests that the specimens described in this contribution display repaired injuries, rather than examples of developmental or genetic aberrations (Owen, 1985; Conway Morris & Jenkins, 1985; Babcock & Robison, 1989; Babcock, 1993; Babcock, 2003; Babcock, 2007; Robison, Babcock & Gunther, 2015; Bicknell & Paterson, 2018; Bicknell, Paterson & Hopkins, 2019; Pates & Bicknell, 2019). The injuries that affect more than one thoracic segment and are located on thoracic areas that are unlikely to have been damaged by chance (Babcock & Robison, 1989; Babcock, 1993; Pates et al., 2017; Bicknell & Paterson, 2018) represent healed injuries, likely attributable to predation. Those injuries showing exoskeletal cicatrisation reflect an attack that occurred recently within the same intermoult period, as observed in modern arthropods (Ludvigsen, 1977; Bursey, 1977; Owen, 1985; Rudkin, 1985; Halcrow & Smith, 1986). Injuries that occurred during a soft-shelled stage, when individuals were likely more vulnerable to attacks, would likely have wrinkled and deformed the exoskeleton as it would not have been fully mineralised (Conway Morris & Jenkins, 1985; Bicknell & Paterson, 2018). Injuries lacking cicatrisation and showing partial regeneration likely occurred in prior moult stages (Owen, 1985; Pates et al., 2017). The SSIs observed in the studied sample may record attacks or moulting complications. In particular, the injuries on *Nevadia weeksi* likely reflect complications during moulting as the most elongated thoracic pleurae that would catch or not exit the old exoskeleton cleanly during ecdysis (Šnajdr, 1978; Owen, 1983; Conway Morris & Jenkins, 1985).

The studied sample presents possible evidence for injury patterns in an entirely qualitative context. This assessment provides potential support for an interpretation of injuries to trilobites caused by predators showing location specificity for the right side; if all injuries do indeed represent failed attacks (e.g., Babcock & Robison, 1989; Babcock, 1993). Six specimens have right-sided injuries, two specimens have potential bilateral injuries and one specimen has a left-sided injury. Although this sample size is too small to test for statistical significance, these data align with the thesis that either Cambrian predators attacked prey from the right side, Cambrian prey orientated themselves to have the right side attacked, or a combination of both (Babcock & Robison, 1989; Babcock, 1993; Eaton, 2019). Evidence for lateralised injury patterns in trilobite systems was also recently presented in the Silurian-aged Rochester Shale (Bicknell, Paterson & Hopkins, 2019). Conversely, Pates et al. (2017) and Pates & Bicknell (2019) reported no statistical evidence for this pattern in their studies of individual Cambrian taxa. As Pates & Bicknell (2019) outlined, studies of injury lateralisation that pool data on injuries from different time periods do not allow researchers to distinguish between potential causes of injuries, as the studied taxa are from different deposits. The true taxon-specific palaeoecological signal is therefore masked by variation in temporal and geographical conditions and it is unlikely that they all were under the same predatory selection pressures (Pates et al., 2017; Bicknell, Paterson & Hopkins, 2019; Pates & Bicknell, 2019). The identification of both Cambrian (Babcock, 1993; Eaton, 2019) and Silurian signals (Bicknell, Paterson & Hopkins, 2019), with the failure to detect a Cambrian signal (Pates et al., 2017; Pates & Bicknell, 2019), and post-Cambrian signal in other cases (Babcock, 1993) demonstrates that the causes of injury lateralisation are best considered on a case-by-case basis. Such an approach provides the best chance of identifying the root causes of an interesting ecological interaction.

No cephalic injuries were reported in this study. This rarity of cephalic injuries has been noted by previous workers (e.g., Owen, 1985; Babcock, 1993; Pratt, 1998). Biological explanations for this pattern could be that predators targeted the thorax and pygidium preferentially, a higher mortality rate of injuries to the head region, and/or trilobites protecting the head region through behavioural actions such as enrolment (Ortega Hernández, Esteve & Butterfield, 2013; Bicknell & Paterson, 2018; Pates & Bicknell, 2019). It is unlikely that it represents sampling bias, as specimens have been collated from a large number of collectors, and trilobite cephala and cranidia provide a wide range of taxonomic, morphometric, and phylogenetic characters and landmarks (e.g., Lieberman, 1999; Webster, 2015). Furthermore, in a bulk sample with no collection bias injured cephala were significantly rarer than thoracic injuries (Pates & Bicknell, 2019).

### Potential predators

Injuries could have been produced by either self-injury during moulting, or the action of predators (Owen, 1985; Babcock, 1993). The traditional perspective is that radiodonts were likely the culprits for Cambrian sublethal, healed trilobite injuries (Rudkin, 1979; Nedin, 1999; Leighton, 2011; Zamora et al., 2011). This group of nektonic Paleozoic stem-group euarthropods (Daley et al., 2009) have often been referred to as ‘anomalocaridids’ or ‘anomalocarids’ in reference to the family Anomalocarididae and the first documented radiodont: *Anomalocaris canadensis* Whiteaves, 1892 (Whittington & Briggs, 1985). The raptorial appendages known to anomalocaridids and amplectobeluids have been highlighted as possible tools for grasping, flexing and breaking trilobite exoskeletons (Babcock, 1993; Nedin, 1999). While some sublethal injuries were potentially caused by this group, the shell-crushing (durophagous) effectiveness of appendage morphologies has been questioned (Pratt, 1998; Bicknell & Paterson, 2018). The slender and elongate auxiliary spines in some *Anomalocaris* species (*Anomalocaris magnabasis* Pates et al., in press and *A. saron* Hou, Bergström & Ahlberg, 1995) is not indicative of a purely durophagous feeding mode (Pates et al., in press). Furthermore, Radiodonta are now considered a group of arthropods with a diverse range of ecologies, from raptorial predation in Anomalocarididae and Amplectobeluidae (e.g., Daley & Edgecombe, 2014; Liu et al., 2018), to sediment sifting in Hurdiidae (e.g., Daley, Budd & Caron, 2013; Moysiuk & Caron, 2019), and filter feeding in Hurdiidae and Tamisiocarididae (Vinther et al., 2014; Van Roy, Daley & Briggs, 2015; Lerosey-Aubril & Pates, 2018). The diversity of feeding modes is supported by the discovery of multiple radiodonts with different inferred ecologies from the same site (Daley & Budd, 2010; Pates & Daley, 2019; Pates et al., in press).

Analysis of the radiodont oral cone has not provided any definitive evidence to support a durophagous lifestyle for these animals (Whittington & Briggs, 1985; Hou, Bergström & Ahlberg, 1995; Hagadorn, 2009; Hagadorn, Schottenfeld & McGowan, 2010; Daley & Bergström, 2012), despite suggestions that the shape might be suitable for producing ‘W’-shaped injuries (e.g., Babcock & Robison, 1989; Nedin, 1999). These lines of evidence, combined with the lack of any hard-parts in known radiodont guts (e.g., Daley & Edgecombe, 2014), has led to suggestions that radiodonts may not have fed on hard-shelled taxa at all (with some potential exceptions discussed below).

Cambrian-aged trilobites and other artiopodans that display gnathobases on protopodal sections of thoracic appendages were potentially durophagous predators that fed like horseshoe crabs (Babcock, 2003; Bicknell et al., 2018a; Bicknell et al., 2018b; Bicknell et al., 2018c; Bicknell, Pates & Botton, 2018d; Bicknell & Paterson, 2018; Holmes, Paterson & García-Bellido, in press). An example of this is *Sidneyia inexpectans* Walcott, 1911 that is known to have shelly cololites (Bruton, 1981; Zacaï, Vannier & Lerosey-Aubril, 2016; Peel, 2017; Bicknell & Paterson, 2018) and fortified gnathobasic spines for effective durophagy (Bicknell et al., 2018c). This was confirmed with recent 3D biomechanical modelling (Bicknell et al., 2018b). Other possible durophagous predators include *Utahcaris orion* Conway Morris & Robison, 1988 that has also been noted with fragmented sclerites in the gut tract (Conway Morris & Robison, 1988; Babcock, 2003; Legg & Pates, 2017). Beyond these artiopodan groups, amplectobeluid genera *Amplectobelua* Hou, Bergström & Ahlberg, 1995 and *Ramskoeldia* Cong et al., 2018 have been documented with gnathobase-like structures near the mouth (Cong et al., 2017; Cong et al., 2018), and three species of the genus *Caryosyntrips* Daley & Budd, 2010 possess stiff appendages with short and robust spines (Daley & Budd, 2010; Pates & Daley, 2017). This suggests that amplectobeluid radiodonts, and *Caryosyntrips* (currently unassigned to a family) were potentially capable of consuming harder prey. Nonetheless, such ideas need quantitative testing, as done for *S. inexpectans* (Bicknell et al., 2018b).

One final consideration regarding possible predators is the idea that injuries may have been inflicted by shell hammering, as opposed to shell crushing (Pratt, 1998). It has been suggested that raptorial frontal appendages of *Yohoia tenuis* Walcott, 1912 would have been effective at breaking biomineralised exoskeletons, using similar mechanics to modern-day mantis shrimps (Pratt, 1998; Haug et al., 2012; Bicknell & Paterson, 2018). Analyses of such morphologies with comparisons to extant stomatopods may highlight the effectiveness of such Cambrian shell hammering (Crane et al., 2018).

### Escalation and predation

Escalated evolution reflects selective pressure placed on individuals by predators, parasites, competitors and dangerous prey (Vermeij, 1994; Vermeij, 2013). Such pressures drive the development of adaptive features in prey to avoid, escape, or defend against predators (Vermeij, 1994; Vermeij, 2013; Thompson, 1999; Baumiller & Gahn, 2004). The record of prey escalation includes changes to external shell ornamentation, fluctuation in predation intensity, and prey regeneration frequency (Vermeij, Schindel & Zipser, 1981; Kelley & Hansen, 1996; McShea, 1998; Alexander & Dietl, 2001; Baumiller & Gahn, 2004; Whitenack & Herbert, 2015). Vermeij (1989) suggested that escalation was a major component of evolution during the Cambrian Explosion and that escalated predation pressures drove the variety of defensive features in prey (Vermeij, 1989; Bengtson, 2002; Brett & Walker, 2002; Babcock, 2003; Marshall, 2006; Vendrasco et al., 2011; Wood & Zhuravlev, 2012; Voje et al., 2015). However, there is limited quantitative evidence for this evolutionary explanation (Bicknell & Paterson, 2018). The Cambrian escalation event could potentially be demonstrated by documenting changes in defensive adaptations of Cambrian trilobites. To conduct such a study, specimens of the same species from different stratigraphic levels within the same formation could be examined for injuries and responses to predation. If Cambrian trilobites did experienced escalated evolution, innovation in defensive features, such as increased exoskeletal thickness, or changes to hypertrophied spines (Pates & Bicknell, 2019), would be observed, and their role in response to the predation tested. An increased number of injured specimens at particular levels within the section would indicate that a higher survival rate from attacks (Vendrasco et al., 2011). Trilobites, with their excellent fossil record, high diversity, high disparity, abundance, and long record of predation, therefore represent a suitable system for understanding the Cambrian escalation event.

## Conclusions

The current study of abnormal Cambrian trilobites within the Paleontological collection of the Smithsonian National Museum of Natural History presents nine new examples of injured specimens. These injuries display a range of morphologies that are attributed to failed predation and complicated moulting. The possible predatory groups are discussed, and euarthropods with gnathobases and other forms of robust spines are considered as the most probable predators. It is also highlighted that trilobites represent an ideal study system for documenting quantitative evidence for the Cambrian escalation event and responses of prey items to the first durophages.

## Additional Information and Declarations

### Data Availability

The following information was supplied regarding data availability: The raw data are photographs presented in Figs. 1–8. The specimens are housed in the Paleontological collection of the Smithsonian National Museum of Natural History (USNM PAL): *Elliptocephala asaphoides* USNM PAL 18350a, *Mummaspis oblisooculatus* USNM PAL 443790, *Olenellus thompsoni* USNM PAL 729428, *Olenellus getzi* USNM PAL 729422, *Nevadia weeksi* USNM PAL 56792a; USNM PAL 56792d, *Glossopleura gigantea* USNM PAL 729419, *Ogygopsis klotzi* USNM PAL 729421, *Elrathia kingii* USNM PAL 729417.
